# The COVID-19 pandemic: a new challenge for syndromic surveillance

**DOI:** 10.1017/S0950268820001314

**Published:** 2020-06-18

**Authors:** Alex J. Elliot, Sally E. Harcourt, Helen E. Hughes, Paul Loveridge, Roger A. Morbey, Sue Smith, Ana Soriano, Amardeep Bains, Gillian E. Smith, Obaghe Edeghere, Isabel Oliver

**Affiliations:** 1 Real-time Syndromic Surveillance Team, Field Service, National Infection Service, Public Health England, Birmingham, UK; 2 COVID-19 Surveillance Cell, Public Health England, London, UK; 3 National Infection Service, Public Health England, London, UK

**Keywords:** Challenges, coronavirus, COVID-19, pandemic, surveillance, syndromic surveillance

## Abstract

The COVID-19 pandemic is exerting major pressures on society, health and social care services and science. Understanding the progression and current impact of the pandemic is fundamental to planning, management and mitigation of future impact on the population. Surveillance is the core function of any public health system, and a multi-component surveillance system for COVID-19 is essential to understand the burden across the different strata of any health system and the population. Many countries and public health bodies utilise ‘syndromic surveillance’ (using real-time, often non-specific symptom/preliminary diagnosis information collected during routine healthcare provision) to supplement public health surveillance programmes. The current COVID-19 pandemic has revealed a series of unprecedented challenges to syndromic surveillance including: the impact of media reporting during early stages of the pandemic; changes in healthcare-seeking behaviour resulting from government guidance on social distancing and accessing healthcare services; and changes in clinical coding and patient management systems. These have impacted on the presentation of syndromic outputs, with changes in denominators creating challenges for the interpretation of surveillance data. Monitoring changes in healthcare utilisation is key to interpreting COVID-19 surveillance data, which can then be used to better understand the impact of the pandemic on the population. Syndromic surveillance systems have had to adapt to encompass these changes, whilst also innovating by taking opportunities to work with data providers to establish new data feeds and develop new COVID-19 indicators. These developments are supporting the current public health response to COVID-19, and will also be instrumental in the continued and future fight against the disease.

## Background

The 2019 coronavirus pandemic (COVID-19) is currently presenting significant challenges to society, health and social care services and science. Following the early reports in December 2019 of cases of severe pneumonia with unknown aetiology in Wuhan province, China [1], the COVID-19 outbreak has now developed into a global pandemic with an identified causal organism (SARS-CoV-2) [2], and early reports of the epidemiological and clinical characteristics of COVID-19 cases have been published [3]. Within Europe, the United Kingdom (UK) reported some of the earliest recorded importations of COVID-19, with the first cases detected at the end of January 2020 [4, 5]. During March 2020, the UK government introduced a range of measures to limit transmission in the community including guidance (and enforceable measures) advising the public to avoid all non-essential travel (including to places of work) and contact with others outside the home.

The challenges presented to public health organisations have been multi-faceted. Surveillance systems form a mainstay of public health intelligence needed to track the progress of the pandemic at the national, regional and local level. Surveillance provides vital intelligence about the sections of the population most at risk, which may inform interventions to mitigate the progression of disease in the population and address any observed inequalities.

Many countries and public health bodies utilise ‘syndromic surveillance’ (using real-time, often non-specific symptom/preliminary diagnosis information collected during routine healthcare provision) to supplement public health surveillance programmes [6]. Syndromic surveillance can rapidly inform on the impacts of infectious diseases, non-communicable diseases and other threats to the health of the population. Fundamentally, syndromic surveillance systems are designed to augment existing public health surveillance programmes by providing: additional early warning through the collection of symptoms/preliminary diagnoses rather than laboratory confirmed diagnoses; situational awareness during an incident to inform, in real-time, on the progression of the incident in the population; reassurance of lack of impact, which can be particularly useful during mass gatherings.

Published literature highlights the flexibility of using generic symptom-based indicators for monitoring a range of conditions, including newly emerging pathogens. In the past, syndromic surveillance systems have shown their benefit in monitoring both seasonal disease, and responding to major emergencies e.g. the 2009 global influenza pandemic [7–9].

The current COVID-19 pandemic has presented a series of unprecedented challenges to syndromic surveillance. In this short paper we highlight some of these challenges using examples from the recent experience of the Public Health England (PHE) national Real-time Syndromic Surveillance Team [10].

## The challenges for syndromic surveillance

### Impact of media reporting

During the early stages of the COVID-19 pandemic there was a growing public interest in global developments as reported (and widely accessible) through various media channels. Previous work has demonstrated the potential impact of media reporting on healthcare seeking behaviour and its effect on routine healthcare system data used by syndromic surveillance [11]. During COVID-19 this impact has been particularly evident. In the UK, the importation of the first confirmed COVID-19 cases [4], the mass repatriation of UK nationals (and subsequent quarantine in local centres) and early localised outbreaks all generated significant national and local media coverage. The impact was observed in increases in syndromic signals (both nationally and locally), particularly those associated with the symptoms reportedly associated with COVID-19 by the media. However, historical experience of different syndromic surveillance systems revealed that some (e.g. telehealth) are more susceptible to the impact of media reporting and therefore a multi-syndromic system approach (encompassing data on different types and levels of healthcare usage) has been beneficial. The prioritisation of syndromic systems which have historically demonstrated less impact from media reporting provided added confidence to interpretation and messages.

### Social and physical distancing measures impacting on healthcare seeking behaviour

The introduction of national guidance on social distancing has had perhaps one of the most unexpected and dramatic impacts on syndromic surveillance. Calculating incidence rates or presenting syndromic counts as a percentage of total activity has, to date, been vital for understanding community burden: accurate population denominators are key to calculating these metrics. During March 2020, syndromic surveillance systems started to detect changes in the healthcare-seeking behaviour of the general public, with large reductions in the number of emergency department (ED) attendances and general practitioner (GP) consultations, particularly for non-respiratory diseases/conditions [12]. These changes presented challenges for interpreting data on different levels. In certain instances, the fall in denominator affected the overall percentage of certain indicators relative to the total which would have led to misinterpretation of data, for example: the daily percentage of acute respiratory infection attendances in EDs increased dramatically, however, the actual numbers seen were largely unchanged or even slightly lower than in the preceding winter; the percentage of cardiac attendances was stable, with no obvious sign of change in patient presentation, however, the numbers attending for cardiac conditions had decreased by almost 50%, which was of great public health concern.

In this instance, the presentation of ED syndromic surveillance data was changed from ‘percent’ to ‘counts’ to overcome these reporting issues. However, often these changes are not straightforward and require significant work to change underlying reporting systems (and responding to subsequent queries) which, while responding to a pandemic, is challenging. Furthermore, during COVID-19, the responsibility of public health organisations continues to be to ‘protect the population from all public health threats’. Therefore, the multi-hazard approach of syndromic surveillance requires that during COVID-19 the surveillance systems can still detect, respond to and monitor non-COVID-19 health incidents. Changes in denominators and the shifts in the presentation of non-respiratory conditions in healthcare services have tested the ability of the systems to detect other public health threats. For example, statistical aberration detection methods used for syndromic surveillance generally use historical baselines to determine alarm thresholds, however, these systems need to be able to adapt to changes in the current ‘observed’ data to continue to detect unusual signals [13].

### Changes in healthcare systems

Syndromic surveillance systems capture data from healthcare (and non-healthcare) systems without requesting additional information from front-line clinicians, or setting standards for coding or data collection. During the COVID-19 pandemic, change in advice on which health services the public should contact and when, has meant that demands on healthcare services in England have altered. To cope with these changing demands, health services have themselves adapted some of the processes by which patients are managed and treated, and how clinical data are coded and collected. This has presented a major challenge to PHE syndromic surveillance.

During the 2009 influenza pandemic, new clinical codes for ‘swine flu’ were released by healthcare providers, however, within these healthcare systems the new codes were still identifiable as, and readily available for use within existing influenza-like indicators e.g. influenza-like illness (ILI). However, in the UK during the early stages of COVID-19, clinical codes specific for COVID-19 have been released and used for assessing and diagnosing patients presenting with relevant symptoms. PHE syndromic systems and indicators were not set up to capture and monitor these new codes. Furthermore, there was the requirement from public health organisations for COVID-19-specific indicators to be reported separately from other respiratory presentations. The need for real-time adaption and extension of surveillance capabilities for COVID-19 presented a challenge for syndromic surveillance.

Firstly, the new additional COVID-19 clinical codes had to be included within daily data extracts which involves working with external data suppliers to understand the COVID-19 specific clinical codes in use and whether they could be collected using existing syndromic data submission mechanisms. Once new codes could be received, surveillance reporting systems had to be adapted to report on ‘COVID-19-like’ indicators. There was a further complication from the introduction of new codes as some pre-existing syndromic indicators were subsequently affected: indicators dropped across several syndromic systems as patient activity was captured using COVID-19 codes e.g. NHS 111 (telehealth) calls for cough. This presented a further challenge as existing indicators were left difficult to interpret, or rendered unusable.

Other changes observed during COVID-19 have been wholesale changes to the way that the public were told to access healthcare services, and how patients were managed [14]. Although the development and promotion of the public use of new web-based and telehealth triage systems by NHS 111 presented a challenge to capture new COVID-19 activity, it also created an opportunity to establish new and novel feeds of valuable anonymised surveillance data to report on these new triage systems e.g. NHS 111 online completed health assessments [15].

Monitoring changes in healthcare utilisation is key to interpreting COVID-19 surveillance data and understanding the impact of the pandemic in the population; syndromic surveillance can play an important role in this.

### Demand for information

High-profile national public health incidents create a demand for information and data to be rapidly shared. This can be extremely challenging, particularly where the level of transparency requested may fall short of information governance requirements expected in the provision of the data for public health surveillance purposes in addition to not enabling the inclusion of methods/additional intelligence required for interpretation.

There is potential risk that the sharing of raw healthcare service data, without interpretation (based on experience of each individual surveillance system and full knowledge of limitations) may compromise key messages coming from public health organisations, which can lead to confusion. In line with routine seasonal surveillance, PHE syndromic surveillance often works alongside and in support of other surveillance programmes (e.g. respiratory, gastrointestinal), and with COVID-19 this has been no different. Within the incident management structure in PHE, a COVID-19 ‘Surveillance Cell’ provides oversight of all COVID-19 surveillance, enabling a coordinated response to requests for information. Syndromic surveillance should always strive to work in collaboration with others in public health to provide a coordinated and correctly interpreted ‘total picture’, rather than as a stand-alone unit.

## Discussion

On reflection, it is important to consider the differences between two recent global pandemics and the role that syndromic surveillance has played in both. In the UK, during the 2009 influenza pandemic syndromic surveillance played a significant role in providing the necessary public health intelligence on the spread of disease, thus helping with the management of the pandemic, particularly during the later ‘mitigation’ phase: when laboratory testing of pandemic influenza cases was restricted to those complex cases where a laboratory results was needed to influence further medical care.

In contrast, to date, the COVID-19 pandemic has seen a call for unprecedented levels of laboratory testing in the community, particularly after moving from the ‘containment’ to ‘delay’ phase [16]. In comparison to 2009, there has been the reliance in 2020 on laboratory reporting of positive cases as a key statistic used to monitor the progression of COVID-19.

During 2009, community-based syndromic surveillance systems were the mainstay of surveillance during the mitigation phase; during 2020 so far, syndromic surveillance has played an important role in supporting laboratory and mortality reporting.

### A forward look

At the time of writing (May 2020), the UK has passed the peak of activity in the COVID-19 ‘first wave’ as confirmed by a combination of different surveillance streams including syndromic surveillance [17]. PHE has now developed a suite of syndromic surveillance COVID-19 indicators that monitor trends in COVID-19-like disease activity across a range of healthcare services (Table 1) [10]. However, as the pandemic further evolves, the needs of government and health organisations will also develop, and there will likely be further changes in healthcare service provision to meet these needs. Therefore, syndromic surveillance systems will need to continue to adapt to take account of these changes and also meet the demand for surveillance outputs that deliver timely and useful ‘information for action’. This further highlights the importance of those running syndromic systems having close links with the data providers, and thus being able to interpret in real-time the surveillance outputs against a background of significant changes to the healthcare system infrastructure. Without these close links, there would be considerable potential for misinterpretation of surveillance data and misleading subsequent key messages.

**Table 1.** Newly developed COVID-19 syndromic indicators developed for PHE syndromic surveillance systems

Syndromic surveillance system	COVID-19 indicator	Descriptor
NHS 111 (telehealth)	Potential COVID-19 calls (percentage of total calls)	Telephone calls assessing patients with COVID-19 symptoms using specific clinical pathways
NHS 111 (online)	Potential COVID-19 online assessments (number)	Completed online COVID-19 assessments ending with further health advice
Emergency department	COVID-19-like attendances (number)	ED attendances where a COVID-19 SNOMED code is used as the first diagnosis code
General practitioner (GP) in hours	COVID-19-like consultations (rate per 100 000 population)	GP consultations using a SNOMED COVID-19 code
GP out of hours	Existing respiratory indicators	N/A
Ambulance	COVID-19-like ambulance dispatch calls	Calls using specific chief complaints for a pandemic

Furthermore, a key area of current PHE syndromic surveillance development is anticipating future public health needs during COVID-19. The immediate and obvious challenges will be those that we will face during winter 2020/2021. Syndromic surveillance data contribute to the annual programme of influenza and other respiratory pathogens [18], with indicators that are sensitive to, and successfully monitor community-based influenza activity [9, 19, 20]. Indeed, these indicators can also be used to identify and monitor trends in other specific respiratory pathogens e.g. respiratory syncytial virus (RSV) activity is observable in ED presentations for acute bronchiolitis in the youngest age groups [21]. However, during winter 2020/2021 SARS-CoV-2, a new respiratory pathogen, is expected to contribute to the winter pressures, and from the currently available evidence it will present with very similar symptoms to influenza (fever, cough) and RSV (cough). This presents a challenge on several levels: how can we disentangle the impact of influenza, RSV and SARS-CoV-2 using generic respiratory syndromic indicators; and how will this impact on our established programmes of surveillance for influenza and RSV? One of the intrinsic features of syndromic surveillance is the flexibility of available indicators enabling a multi-hazard approach; this will be a strength that will support the future COVID-19 response. It will be important to establish which syndromic indicators (in which syndromic surveillance systems) are most sensitive to COVID-19, or which indicator/age group or symptom combinations provide the best means to disentangle COVID-19 from other respiratory diseases. PHE are currently planning the surveillance programme for winter 2020/2021 and how to meet these challenges: syndromic surveillance will form a core of these plans.

Across Europe, the Moving Epidemic Method (MEM) threshold has been adopted for monitoring seasonal influenza activity, including early warning and intensity [22]. These thresholds provide public health organisations with a simple indication of the level of influenza, which then influences messaging and the application of policy. The MEM thresholds are often based upon syndromic indicators e.g. GP consultations for influenza-like illness. These in turn are based upon collections of codes used by clinicians to categorise diagnoses/symptoms; an additional contribution from COVID-19 to ILI presentations could make existing influenza activity thresholds difficult to interpret and inaccurate in measuring community-based influenza activity.

Syndromic surveillance has traditionally contributed to the surveillance of infectious diseases. However, the COVID-19 pandemic has illustrated the importance that syndromic surveillance is having in understanding the wider impact of the pandemic on health, not just COVID-19. These systems are showing the potential for identifying changes in healthcare seeking behaviour that can influence national guidance and public health messaging. Decreases in ED attendances highlighted a possible adverse impact of the social distancing phase. During this phase, the population was advised to reduce physical contact with healthcare services (e.g. use telephone consultations), and there might also have been a reluctance to expose themselves to the healthcare setting. Patients needing urgent care for non-respiratory conditions were not seeking healthcare in EDs. This led to concern about later surges in demand as these conditions worsened, and possible increases in excess (non-COVID-19) deaths, leading to further public health messaging urging the public to seek healthcare advice swiftly for more severe conditions e.g. myocardial ischaemia and strokes [23]. However, decreases in other indicators of infectious disease presentations, such as gastroenteritis, were also noted, possibly indicating a true decrease in disease incidence in the community.

The current focus of syndromic surveillance has been on monitoring the progression of the pandemic at country level to inform the national response. However, there is an opportunity to explore how the needs of the local public health response can be supported with these data. While syndromic surveillance data are generally population level-based (not individual cases), and with some national systems not having full population coverage (i.e. sentinel), providing syndromic surveillance intelligence at the local level can be challenging. However, it is important to explore the utilisation of syndromic data for the local level response as these data could play a central role in being able to identify local resurgence of the disease to inform local control measures.

Finally, syndromic surveillance has previously been shown to play an important role in the evaluation of the impact of the introduction of new vaccination programmes [24, 25]. Therefore, if a future COVID-19 vaccine is developed and reaches the stage of wide use in the population, syndromic surveillance systems may play an important role in assessing the overall impact of such vaccine(s) by monitoring reductions in community-based respiratory infections.

## Data Availability

Restrictions apply to the availability of the data that support the findings of this study. Surveillance outputs and limited data are routinely available at https://www.gov.uk/government/collections/syndromic-surveillance-systems-and-analyses and can be used acknowledging the source.
